# Effects of Polyamines on *Vibrio cholerae* Virulence Properties

**DOI:** 10.1371/journal.pone.0060765

**Published:** 2013-04-10

**Authors:** John Bradley Goforth, Nicholas Emmanuel Walter, Ece Karatan

**Affiliations:** Department of Biology, Appalachian State University, Boone, North Carolina, United States of America; East Carolina University School of Medicine, United States of America

## Abstract

*Vibrio cholerae* is the causative agent of the severe enteric disease cholera. To cause cholera the bacterium must be able to synthesize both cholera toxin (CT) and toxin-coregulated pilus (TCP) which mediates autoagglutination and is required for colonization of the small intestine. Only a few environmental signals have been shown to regulate *V. cholerae* virulence gene expression. Polyamines, which are ubiquitous in nature, and have been implicated in regulating virulence gene expression in other bacteria, have not been extensively studied for their effect on *V. cholerae* virulence properties. The objective of this study was to test the effect of several polyamines that are abundant in the human intestine on *V. cholerae* virulence properties. All of the polyamines tested inhibited autoagglutination of *V. cholerae* O1 classical strain in a concentration dependent manner. Putrescine and cadaverine decreased the synthesis of the major pilin subunit, TcpA, spermidine increased its production, and spermine had no effect. Putrescine and spermidine led to a decrease and increase, respectively, on the relative abundance of TCP found on the cell surface. Spermine led to a small reduction in cholera toxin synthesis whereas none of the other polyamines had an effect. The polyamines did not affect pili bundling morphology, but caused a small reduction in CTXφ transduction, indicating that the TCP present on the cell surface may not be fully functional. We hypothesize the inhibition of autoagglutination is likely to be caused by the positively charged amine groups on the polyamines electrostatically disrupting the pili-pili interactions which mediate autoagglutination. Our results implicate that polyamines may have a protective function against colonization of the small intestine by *V. cholerae.*

## Introduction

*Vibrio cholerae*, a Gram-negative, enteropathogenic organism, is the causative agent of the disease cholera. Cholera is a severe and life threatening diarrheal disease, with a high mortality rate in regions without potable drinking water. Areas with poor sanitation and access to potable drinking water are at high risk for epidemic outbreaks due to the oral-fecal transmission route of the bacterium. Currently, *V. cholerae* is classified into over 200 distinct serogroups based upon the differences in the sugar composition of the “O” antigen present on the bacterial surface [1], [2]. Of these only O1, subdivided into classical and El Tor biotypes, and O139 are capable of causing epidemic cholera.

Cholera occurs when an infectious dose of *V. cholerae* is orally ingested, and the bacteria subsequently colonize the small intestine [3], [4], [5]. Inside the host, *V. cholerae* synthesizes two main virulence factors, cholera toxin (CT) and toxin-coregulated pilus (TCP). CT is an enterotoxin secreted by the bacterium that causes the characteristic voluminous diarrhea [6], [7] and TCP aids in the colonization of the small intestine [6], [8]. In addition to TCP and CT, *V. cholerae* synthesizes other virulence factors which contribute to pathogenesis; however, when either TCP and/or CT synthesis is reduced, or absent, colonization and virulence are markedly attenuated [6].

TCP belongs to the Type IVb class of pili, which are involved in pathogenesis of other organisms as well, including *Escherichia coli* and *Neiserria gonorrhoeae* [9], [10], [11]. TCP is composed of a repeating homopolymer of the major pilin subunit, TcpA, which forms a helical arrangement originating in the inner membrane and protruding outward from the cell surface [12], [13]. The genes which code for the proteins making up the TCP biogenesis apparatus, and the major pilin subunit are located within the *tcp* operon found on a segment of the chromosome referred to as the *Vibrio* pathogenicity island [14]. Colonization and autoagglutination resulting in microcolony formation within the small intestine has been shown to be mediated by TCP [6], [9], [12], [15], [16], [17]. Autoagglutination can be assessed *in vitro* by culturing bacteria under optimal TCP expressing conditions [8], [15]. *In vitro* autoagglutination has been shown to correlate well with colonization in the infant mouse model of cholera, indicating the reliability of *in vitro* autoagglutination as a positive indicator of *V. cholerae* to effectively colonize the host [15].

The regulation of *V. cholerae* virulence genes is controlled through a complex pathway consisting of multiple proteins, each playing an integral role in the regulation of both *ctxAB* genes encoding the two subunits of CT, and the *tcp* operon [18]. Virulence gene expression has been shown to respond to specific environmental stimuli *in vitro,* including temperature, pH, bile salts, osmolarity, bicarbonate and the presence of certain amino acids [19], [20], [21], [22]. Interestingly, the conditions required to promote maximal virulence gene expression *in vitro* are different than those encountered within the small intestine. Conditions for maximal virulence gene expression *in vitro* for *V. cholerae* O1 classical biotype were found to be a temperature of 30°C, pH 6.5 and a salt concentration of approximately 66 mM NaCl [23]. Conversely, environmental conditions within the small intestine are a temperature of 37°C, a higher pH and an osmolarity equivalent to approximately 300 mM NaCl [23]. Within the intestinal environment only a few signals have been identified which regulate virulence gene expression. Animal studies have shown that wild-type *V. cholerae* preferentially colonize the middle/distal portion of the small intestine in a TCP-dependent manner, indicating a potential gradient of either an attractant or repellent leading to the area of colonization [24], [25]. The purpose for this temporal/proximal colonization and virulence gene expression at the more distal portion of the small intestine has not been elucidated.

**Figure 1:**
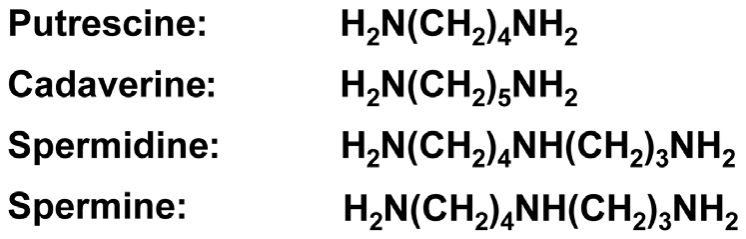
Polyamines used in this study.

There are a plethora of potential signals within the small intestine which have yet to be studied for their effect on *V. cholerae* virulence properties. One group of molecules present in this environment is polyamines, flexible hydrocarbon chains with interspersed amine groups that are positively charged at neutral pH (Fig. 1). These small molecules are ubiquitous in nature and are utilized by almost all living cells for normal cell proliferation [26], [27], [28]. Polyamines are found in millimolar quantities in cells and due to their cationic nature, polyamines interact with RNA, DNA and proteins to modulate various cellular processes [26], [27], [28], [29], [30], [31]. Ability to synthesize, transport, and/or respond to environmental polyamines are important for virulence for a number of bacteria including *Streptococcus pneumoniae*, *Francisella tularensis, Salmonella enterica* Serovar Typhimurium, and *Yersinia pestis* [32], [33], [34], [35]. Also, the polyamines spermidine and putrescine have previously been shown to attenuate MR/K hemagglutination, mediated by type 3 fimbriae, in several members of *Enterobacteriaceae* by a yet unidentified mechanism [36]. *V. cholerae* O1 El Tor mutants lacking the ability to synthesize norspermidine, one of the major polyamines produced by this organism, are not attenuated in the infant mouse model of cholera [37]. However, exogenous polyamines, more commonly found in the host environment, have not been studied for their effects on virulence properties of *V. cholerae.*

Polyamines within the human gastrointestinal tract originate both endogenously and exogenously by means of three independent sources: polyamines which are synthesized by our own cells, those contributed by intestinal microbiota and those from dietary intake [28], [38], [39]. Due to the high proliferation rate of epithelial cells in the gastrointestinal tract, the gut inherently has a high demand for polyamines and thus a relatively high concentration of polyamines compared to other tissues in the body [28], [29], [31]. The polyamines spermidine, spermine, putrescine, and cadaverine are the most abundant in the human intestine [28]. Almost all cells are capable of *de novo* synthesis of the polyamines spermidine and putrescine, with limited exceptions; spermine synthesis is mainly confined to eukaryotic cells and cadaverine synthesis is mainly present in prokaryotic cells [28], [40], [41]. The largest portion of exogenous polyamines in the gastrointestinal tract comes from our diet [28]. The average human diet supplies the body with hundreds of micromoles of spermidine, spermine and putrescine per day [28]. In addition, members of the intestinal microbiota are believed to be the major contributors of polyamines, especially cadaverine, in the distal portion of the gastrointestinal tract [42], [43].

*V. cholerae* colonizing the small intestine are likely to encounter the four previously mentioned polyamines; therefore, it is possible that they play a role in cholera pathogenesis. Thus, the goal of this study was to explore whether these polyamines have an effect on *V. cholerae* virulence properties. We show that at high concentrations all of the polyamines tested negatively influence autoagglutination of *V. cholerae* O1 classical strain *in vitro*, suggesting these host-derived molecules could modulate colonization of the intestine by this bacterium.

## Materials and Methods

### Bacterial Strains, Media and Reagents

Bacterial strains used in this study were *V. cholerae* O1 classical biotype O395 and CL101 (*V. cholerae* O395 containing pCTX-Knφ). *V. cholerae* strain O395 was grown in 1% tryptone broth (1% (w/v) Tryptone, 66 mM NaCl), pH 8.5, with shaking at 200 rpm at 37°C. For the induction of the virulence genes, *V. cholerae* O395 was grown in 1% tryptone broth, pH 6.5 with shaking at 150 rpm at 30°C [19]. Streptomycin (Sm) and Kanamycin (Kn) were used at 100 µg/ml and 50 µg/ml, respectively. Strains were stored at −80°C in Luria Bertani (LB) broth supplemented with 15% glycerol. Spermidine trihydrochloride, putrescine dihydrochloride, spermine tetrahydrochloride, and cadaverine dihydrochloride were from Sigma-Aldrich (St. Louis, M.O.). Polyamine salts were dissolved at a concentration of 1 M in water and prepared fresh prior to each experiment.

### Agglutination Assay

*V. cholerae* O1 classical strain O395 was streaked on LB (Sm) plates and incubated at 37°C overnight. Single colonies were inoculated into 1% tryptone broth, with appropriate antibiotics and incubated overnight in a shaking incubator (220 rpm) at 37°C. The next day, cultures were diluted 1/100 into 2 ml 1% tryptone broth containing streptomycin and incubated in a shaking incubator (250 rpm) at 37°C to mid-log phase. These were then inoculated at a 1∶10,000 dilution into 20 ml of 1% tryptone broth at either pH 6.5 or pH 8.5 and incubated for 18 hours, at 30°C and 37°C, respectively. Following this incubation, 2 ml aliquots were removed from the cultures, in triplicate, briefly vortexed, and optical densities were measured at 595 nm. Cultures were allowed to sediment for 1 hour, and final optical density measurements were taken. Autoagglutination was quantified by assigning a sedimentation index (SI) to all the cultures (SI = 1– [Final A_595_/Initial A_595_]). Where relevant, the media were supplemented with various amounts of the polyamine stock solutions. The concentration of each polyamine added to the media was adjusted such that equal numbers of moles of positive charges were introduced into the media for each of the three concentrations. For example, at the lowest concentration, the diamines, spermidine, and spermidine were used at 15, 10, and 7.4 mM concentrations; however, all of the polyamines contributed approximately 30 mM of positive charges into the media. To assess the effect of a mixture of all of the polyamines, they were added to the media such that the total amount positive charges were 0.6, 1.2, 1.8 mmoles. To ensure reproducibility, the assays were performed with at least three biological replicates in triplicate.

### Osmolarity Calculations

Osmolarity was calculated as follows: molar concentration of solute* number of solutes* osmotic coefficient. Tryptone broth contains 66 mM NaCl. The osmolarity of this broth was calculated using 0.93 as the osmotic coefficient of NaCl [44]. The osmotic coefficients for the polyamine salts are unknown; therefore, an osmotic coefficient of 1 was used in calculations, which would indicate a 100% dissociation of the compound. Therefore, the actual osmolarities of the media containing the polyamines may be lower than the numbers calculated here.

### O395 CTXφKn Transduction Assay

Transduction assays were performed as described [15]. Briefly, *V. cholerae* strain CL101 was streaked onto LB (Kn) agar plates and incubated at 37°C overnight. A single colony was inoculated into 2 ml LB broth containing streptomycin and incubated in a rotating incubator (220 rpm) for 16 hours at 37°C. Bacterial cells were pelleted and the supernatant was filtered through a 0.2 µm syringe filter to remove any residual bacteria. Equal volumes of filtered supernatant containing CTXφKn, and O395 cultures grown under virulence inducing conditions in the presence and absence of polyamines were mixed and incubated at room temperature for 30 minutes. Following the 30 minute incubation, cultures were serially diluted and plated on both LB (Sm) agar plates for total cell input, and LB (Kn) plates for cells transduced, and incubated for 16 hours at 37°C. Transduction frequency was measured as the ratio of Kn^r^ colonies to the number of Sm^r^ colonies. Transduction efficiency was calculated as the ratio of the transduction frequency of the culture containing polyamines to the transduction frequency of the culture that did not. For statistical analysis, transduction assays were performed with three biological replicates in triplicate.

### SDS-PAGE and Immunoblotting

One milliliter aliquots were removed from bacterial cultures and pelleted. The supernatant was transferred to a fresh microfuge tube for cholera toxin detection (described below), and bacterial cells were resuspended in 500 µl 1X phosphate buffered saline (PBS). Whole cell extracts were obtained by sonication of bacterial suspension for 10 seconds at 30% power, duty cycle 40, output 4 using a Heat Systems – Inc. Ultrasonic Processor (Farmingdale, N.Y.), and followed by centrifugation at 16,100×g for 20 minutes at 4°C. Protein concentrations were quantified by Bradford assay using the Coomassie Protein Assay Reagent (Thermo Scientific). Three micrograms of whole cell extracts were separated by SDS-PAGE and blotted onto a PVDF membrane using a semi-dry transfer cell (BioRad) at 15 volts for 15 minutes. The membrane was blocked overnight in 100 ml 5% (w/v) non-fat dry milk in 1X PBST (0.1% (v/v) Tween 20 solution in 1X PBS) at 4°C. The membrane was then incubated with a rabbit monoclonal anti-TcpA antibody, at a 1∶50,000 dilution in 10 ml 1X PBST for 1 hour with constant rotation, followed immediately by three consecutive 1X PBST washes for 5 minutes each. Next, the membrane was incubated with an HRP-conjugated goat anti-rabbit antibody at a 1∶10,000 dilution in 10 ml 1X PBST for 1 hour followed by three subsequent 1X PBST washes for 5 minutes each. To detect the loading control, the blots were incubated with a mouse antibody against the α-subunit of *Escherichia coli* RNA polymerase (Neoclone, Madison, WI.) used at 1∶10,000 dilution and the goat anti-mouse secondary antibody was used at 1∶20,000 dilution. The membrane was then incubated with SuperSignal® West Pico Chemiluminescent Substrate (Thermo Scientific) for approximately 5 minutes, exposed to X-ray film and developed in a Konica Minolta SRX-101A developer (Konica Minolta Medical & Graphic, Inc., China). Densitometry was performed using Image J (http://imagej.nih.gov/ij/) and the measurements were normalized to loading controls. For visual representation, the images were adjusted to 49% of their original size and cropped to assemble a collage.

### Whole Cell ELISA

Experimental procedure was followed as previously described with minor modifications [45]. Briefly, 5 ml of cultures grown under virulence inducing conditions in the presence and absence of polyamines were pelleted; cells were resuspended in 500 µl 1X PBS and vortexed vigorously for 1 minute. Microcolonies which were not dispersed by vortexing were pelleted by centrifugation at 300×g for 2 minutes. Four hundred microliters of the top layer was removed and placed in a fresh microcentrifuge tube. Optical densities of 100 µl of the samples were measured using a Bio-Rad model 680 microplate reader at 595 nm. To correct for differences in optical densities, equivalent amounts of cells were removed from each culture. Cells were resuspended in 250 µl 1X PBS at an optical density of 0.1 and aliquoted into 0.4 ml Nunc 96-well Flat Bottom Immuno Plates (Thermo Scientific). Aliquots were serially diluted 1∶5 into 200 µl 1X PBS for the remaining wells and incubated at 37°C for 1 hour. Wells were washed three times with 1X PBST for 5 minutes each, blocked with 200 µl 5% (w/v) Bovine Serum Albumin (BSA) in 1X PBS and incubated at 37°C for 1 hour. Wells were washed as described above, incubated at 37°C for 1 hour with anti-TcpA antibody diluted 1∶5,000 in 1X PBS, washed again, and incubated at 37°C for 1 hour with HRP-conjugated goat anti-rabbit antibody diluted 1∶5,000 in 1X PBS. Wells were again washed as previously described, followed by a single 1X PBS wash for 5 minutes. Following the removal of the final 1X PBS wash, 100 µl TMB Super Sensitive One Component HRP Microwell Substrate (SurModics®, Eden Prairie, M.N.) were added and the plate was incubated at room temperature approximately 5 minutes. Reactions were stopped by the addition of 100 µl 1 N HCl. Absorbances were measured at A_415_ for wells using a microplate reader. Assays were performed in triplicate with three biological replicates for statistical analysis.

### Cholera Toxin ELISA

One ml aliquots were removed from cultures grown under virulence inducing conditions in the presence and absence of polyamines and pelleted. The supernatant was transferred to a fresh microfuge tube and stored at −20°C until used for cholera toxin detection. Nunc 96-well Flat Bottom Immuno Plates were coated with 100 µl of 2 µg/ml Monosialoganglioside GM_1_ (Sigma-Aldrich) and incubated at room temperature for 4 hours. Following incubation, plates were blotted on an absorbent cloth to remove liquid. Wells were washed three times with 1X PBST for 5 minutes each. The wells were blocked with 200 µl binding buffer (1X PBS, 0.05% (v/v) Tween 20, 0.5% (w/v) BSA) and incubated at room temperature for 1 hour. Liquid was removed and wells were washed as previously described. Supernatants were diluted 1∶10 in 1X PBS, 100 µl of these were added to the wells in quadruplicate and the plates were incubated at room temperature for 1 hour. Wells were washed, 100 µl of anti-CtxB antibody (Abcam, Cambridge, M.A.), diluted 1∶5,000 in binding buffer, were added to each well, and the plates were then incubated at room temperature for 1 hour. Plates were washed again and incubated at room temperature for 1 hour with an HRP conjugated goat anti-rabbit antibody diluted 1∶20,000 in binding buffer. Following the removal of the liquid and washing of wells, enzymatic detection was performed as described above for whole cell ELISA.

### Transmission Electron Microscopy

Five microliter aliquots were removed from bacterial cultures and placed on parafilm. Three hundred-mesh formvar coated copper grids (Ted Pella Inc., Redding, C.A.) were inverted on top of the 5 µl aliquots for 5 minutes to adsorb bacteria to the grids. Remaining liquid was wicked from grids using VWR Light-Duty Tissue Wipers (VWR, West Chester, P.A.) and the grids were washed by inverting onto 5 µl sterile deionized water for 10 seconds. Grids were negatively stained for 5 minutes by inverting onto 5 µl filter sterilized 1% Phosphotungstic acid (EMD, Gibbstown, N.J.) at pH 7. Remaining stain was wicked from the grids and the grids were allowed to air dry at room temperature for 2 days. Grids were imaged in a JEM-1400 S/TEM (JEOL Inc., U.S.A.) at 120 keV.

## Results

### Polyamines Inhibit Autoagglutination of *V. cholerae*

**Figure 2:**
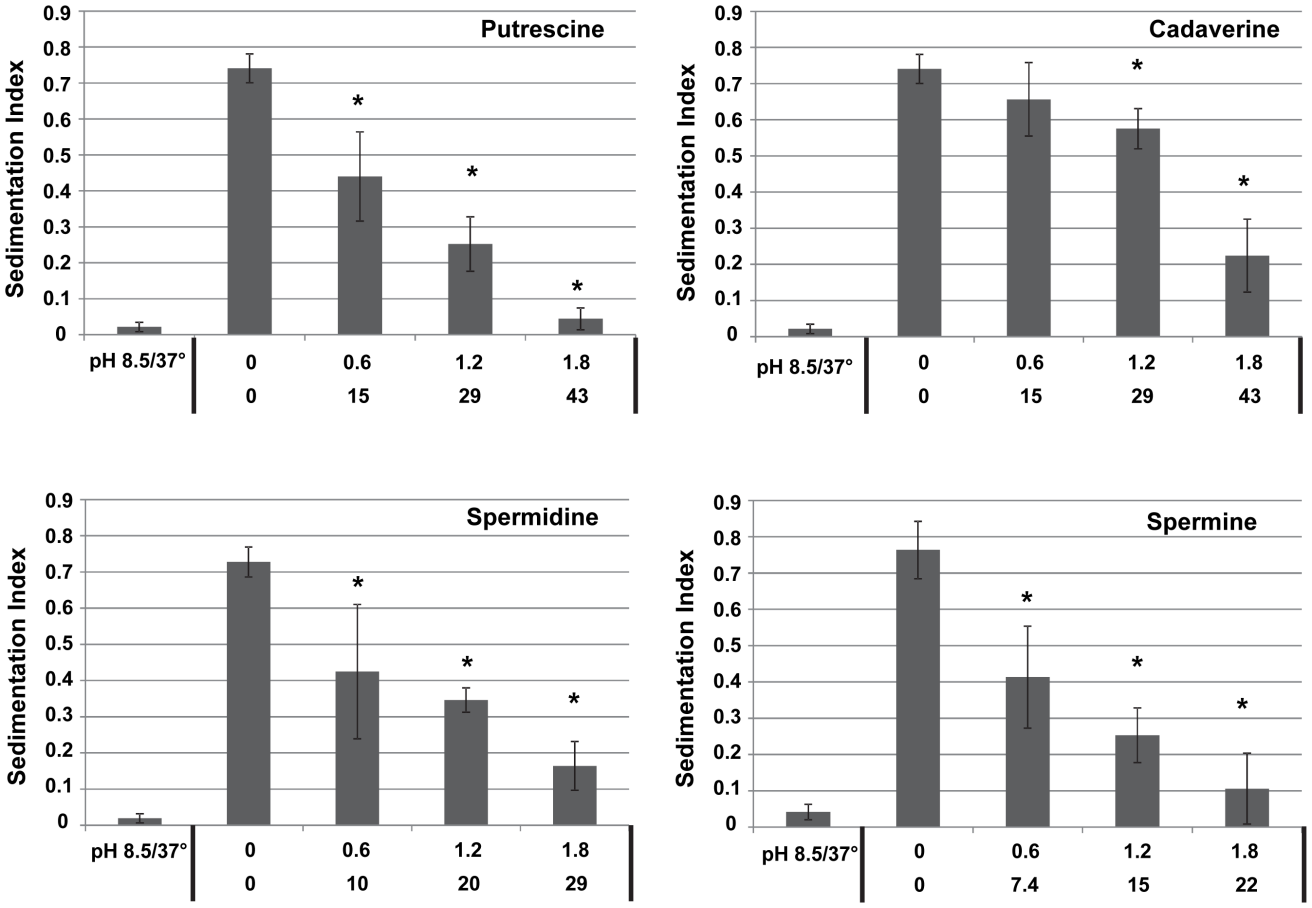
Effect of polyamines on *V. cholerae* autoagglutination. *V. cholerae* was cultured in the presence of increasing concentrations of putrescine, cadaverine, spermidine and spermine under virulence factor inducing conditions. Negative controls were grown in media with a pH of 8.5 at 37°C. For the experimental cultures, the first row of values is mmoles of polyamines added to the media as a result of polyamine supplementation; the second row of values is the molarity of the polyamines in the culture. Sedimentation index was calculated as 1– (Final A_595_/Initial A_595_). Each sample is the average of three biological replicates with error bars representing the standard deviation. Pair-wise Student’s t-tests were performed to compare SI’s of cultures grown in the absence of polyamines to cultures grown with each different polyamine concentration. Stars indicate p<0.01).

Certain strains of *V. cholerae* cultured under virulence factor inducing conditions begin synthesizing TCP which leads the cells to autoagglutinate and form microcolonies. Since microcolonies are heavier, they sediment to the bottom of the culture tube leaving any cells which did not join the microcolonies suspended in the media. A relative quantification of autoagglutination can be achieved by measuring the absorbance of the media before and after sedimentation of the microcolonies in the media. We initially wanted to perform the autoagglutination experiments with *V. cholerae* O1 classical and El Tor biotypes, as well as *V. cholerae* O139. However, we observed autoagglutination only in the *V. cholerae* O1 classical biotypes; therefore, we used this strain in all of our experiments. In order to assess the effect of polyamines on TCP production, agglutination assays were performed with *V. cholerae* cultures grown in the presence and absence of polyamines. The diamines putrescine and cadaverine, the triamine spermidine, and the tetraamine spermine were used in these experiments as these are the most abundant polyamines found in the human intestine [28]. Diamines, triamines, and tetraamines contain 2, 3, or 4 positive charges, respectively, at the pH these experiments were conducted. We hypothesized that the positive charges may have an effect on autoagglutination; therefore, we chose to normalize the amount of polyamines used to the amount of positive charges introduced into the media. For this reason, polyamines were added to introduce 0.6, 1.2, and 1.8 mmoles of positive charges to the media. All of the polyamines tested led to a statistically significant inhibition of autoagglutination in a concentration dependent manner (Fig. 2). Putrescine, spermidine, and spermine had similar effects on autoagglutination whereas cadaverine had the weakest effect on agglutination. For example, presence of 1.2 mmoles of positive charges introduced with cadaverine addition led to only a small reduction in agglutination (22%) whereas the same amount of positive charges resulting from putrescine, spermidine, spermine led to larger decreases in agglutination (70%, 52%, 67%, respectively). To determine if combination of all four polyamines had a similar effect on autoagglutination, assays were done with all four of the polyamines combined in equal amounts (Figure S1). To our surprise, the polyamine mixture was not as effective as putrescine, spermidine, and spermine used along in reducing autoagglutination.

### Putrescine and Cadaverine Lead to Decreases Whereas Spermidine Leads to an Increase in TcpA Synthesis

**Figure 3:**
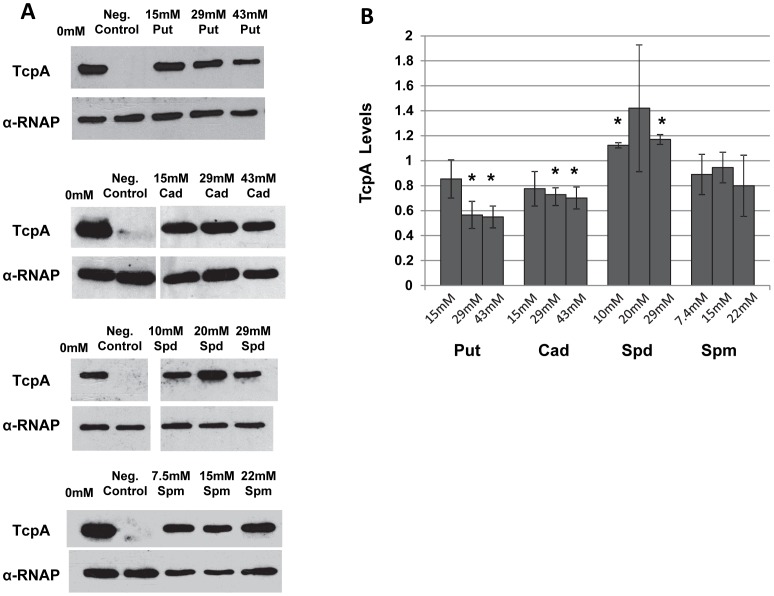
Effect of polyamines on TcpA levels. A. Western blots. Extracts of cells grown in the absence or presence of polyamines were separated by SDS-PAGE, blotted on PVDF membrane, and TcpA was detected using an anti-TcpA antibody. Negative controls were grown in media with a pH of 8.5 at 37°C. The loading control was probed using an antibody against the α-subunit of the *E. coli* RNA polymerase. Although the α-subunit of the *E. coli* RNA polymerase has a higher molecular weight (36 kD) than TcpA (20.5 kD), the images were inverted vertically to aid ease of comparison. Blots shown are representatives of three different experiments performed with three biological replicates. The vertical breaks in the gel images indicate that the lanes in which the cell extracts from cultures grown with cadaverine were not immediately next to the negative control lane. B. Quantification of TcpA levels. TcpA band densities were normalized to the loading control and then compared to those obtained from cells grown without polyamines. TcpA levels are calculated as: normalized band densities in the presence of polyamines/normalized band densities in the absence of polyamines. Each sample is the average of three biological replicates with error bars representing the standard deviation. Pair-wise Student’s t-tests were performed to compare SI’s of cultures grown in the absence of polyamines to cultures grown with each different polyamine concentration. Stars indicate p<0.01. Put: putrescine, Cad: cadaverine, Spd: spermidine, Spm: spermine.

We next wanted to determine whether polyamines inhibited agglutination by decreasing the synthesis of the major pilin subunit, TcpA. Like many type IV pili, TCP is composed of a major pilin subunit that is incorporated into a growing pilus filament held together by hydrophobic and electrostatic interactions [13]. Since polyamines have been shown to bind to DNA, RNA and ribosomes in the cell, it is plausible these molecules inhibit TcpA synthesis directly or indirectly at the transcriptional or translational level. Western blot analysis of whole cell extracts showed that at the highest concentration putrescine and cadaverine led to approximately 50% and a 30% reduction, respectively, in TcpA levels. Spermidine led to a small but statistically significant increase at the highest concentration and spermine had no effect (Fig. 3). These results suggest that spermidine and spermine affect agglutination by a mechanism that does not involve reduction of TcpA production, whereas the effect of putrescine and cadaverine on autoagglutination can be partially explained by their effect on TcpA levels.

One possible explanation for the varying effects polyamines had on TcpA production could be based on osmolarity changes. *V. cholerae* virulence gene expression is extremely sensitive to osmolarity. In one study, raising the osmolarity of the media from 123 mOsm (66 mM NaCl) to 246 mOsm (132 mM NaCl) led to a marked reduction in both CT and TcpA production [46]. To determine if the reduction in TcpA levels caused by putrescine and cadaverine could be explained by increases in osmolarity, we calculated the osmolarity of the media used in agglutination assays (Table 1). Putrescine and cadaverine led to a reduction of TcpA production at approximately 216 mOsm, whereas neither spermidine nor spermine had the same effect at even higher osmolarities of 245 and 239 mOsm, respectively. These results suggest that increases in osmolarity due to the polyamines are not likely to account for the decrease in TcpA production.

**Table 1:** Effect of polyamines CT production, CTX phage transduction, and osmolarity.

Culture Conditions	CT	TE	Osmolarity (mOsm)
No polyamines	1	1	123
15 mM Put	1.00±0.00	−	174
29 mM Put	0.99±0.01	−	216
43 mM Put	0.97±0.01	0.21±0.26	256
15 mM Cad	0.97±0.01	−	174
29 mM Cad	0.97±0.02		216
43 mM Cad	0.98±0.01	0.23±0.11	256
10 mM Spd	0.99±0.02	−	170
29 mM Spd	0.97±0.02	−	208
30 mM Spd	0.99±0.02	0.20±0.11	245
7.4 mM Spm	0.91±0.02	−	168
15 mM Spm	0.91±0.01	−	204
22 mM Spm	0.88±0.02	0.31±0.15	239

Numbers are the average of 3 biological replicates +/− standard deviation. Values were normalized to those obtained in the absence of polyamines, which are reported as “1″ TE:Transduction efficiency, CT: cholera toxin, Put: putrescine, Cad:cadaverine, Spd: spermidine, Spm:spermine.

### Putrescine and Spermidine have Opposite Effects on TCP Biogenesis

**Figure 4:**
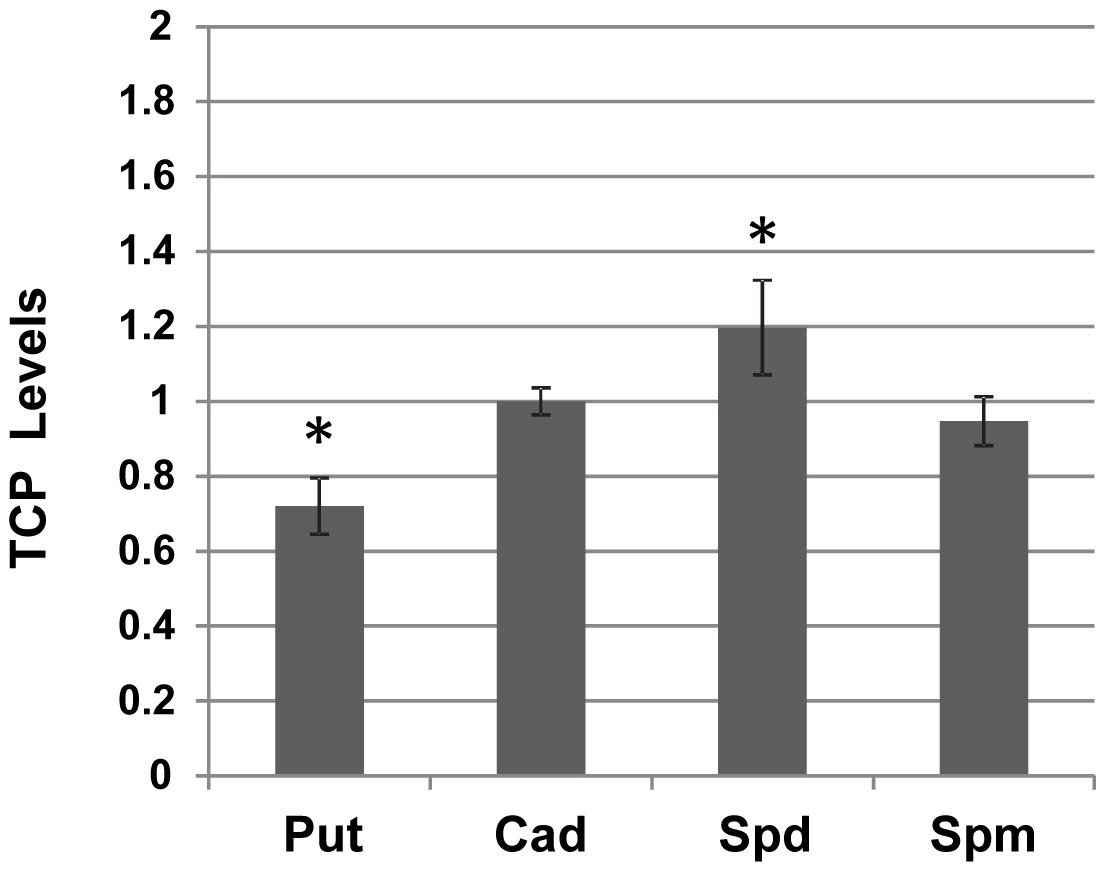
Effect of polyamines on cell-associated TCP levels. Cell-associated TCP was quantified using a whole-cell ELISA as described in Materials and Methods. Values are reported as TCP Levels and were obtained using the following formula: A_420_ with polyamines/A_420_ without polyamines. Each sample is the average of three biological replicates with error bars representing the standard deviation. Pair-wise Student’s t-tests were performed to compare SI’s of cultures grown in the absence of polyamines to cultures grown with each different polyamine Stars indicate p<0.01. Put: putrescine, Cad: cadaverine, Spd: spermidine, Spm: spermine.

We next wanted to determine if the polyamines affected agglutination by disrupting the biogenesis of TCP. To quantify cell-associated TCP, a whole cell ELISA assay was performed in the presence of polyamines at the concentrations that produced the maximum effect on agglutination (Fig. 4). Putrescine led to an approximately 25% reduction in surface-associated TCP compared to cultures grown in the absence of polyamines. Interestingly, cadaverine did not have a significant effect on TCP biogenesis even though TcpA levels were found to be reduced by this polyamine. Spermidine led to an approximately 25% increase in cell-associated TCP levels, which was consistent with its effect on TcpA production. Spermine did not influence the amount of TCP present on the cell surface. These results show that cells are able to synthesize TCP filaments in the presence of spermine and cadaverine at levels comparable to cultures grown in the absence of polyamines, and thus they are not likely to affect the TCP biogenesis apparatus, whereas putrescine and spermidine have opposite effects on the levels on TCP biogenesis.

### Spermine Leads to a Small Reduction of Cholera Toxin Synthesis

In *V. cholerae*, transcription of the *tcp* and *ctx* operons is under the control of the transcriptional regulator, ToxT, leading to the coordinated production of TCP and CT under most conditions [18]. Because putrescine, cadaverine, and spermidine had an effect on cellular TcpA levels, we hypothesized that they may also affect cholera toxin production. Levels of cholera toxin in the culture medium of cells grown in the presence and absence of polyamines were assessed using an ELISA assay. The results showed that putrescine, cadaverine, and spermidine did not have a significant effect on cholera toxin synthesis (Table 1); spermine on the other hand, had a small (approximately 10%), but statistically significant (P<0.0001) inhibition on CT production. However, this effect was seen in all cultures and did not show dose-dependency. These results suggest that TcpA and CT production are not co-regulated by polyamines under the conditions of our experiments.

### Polyamines do not Affect Pilus Morphology or Bundling but do Affect Pilus Functionality

**Figure 5:**
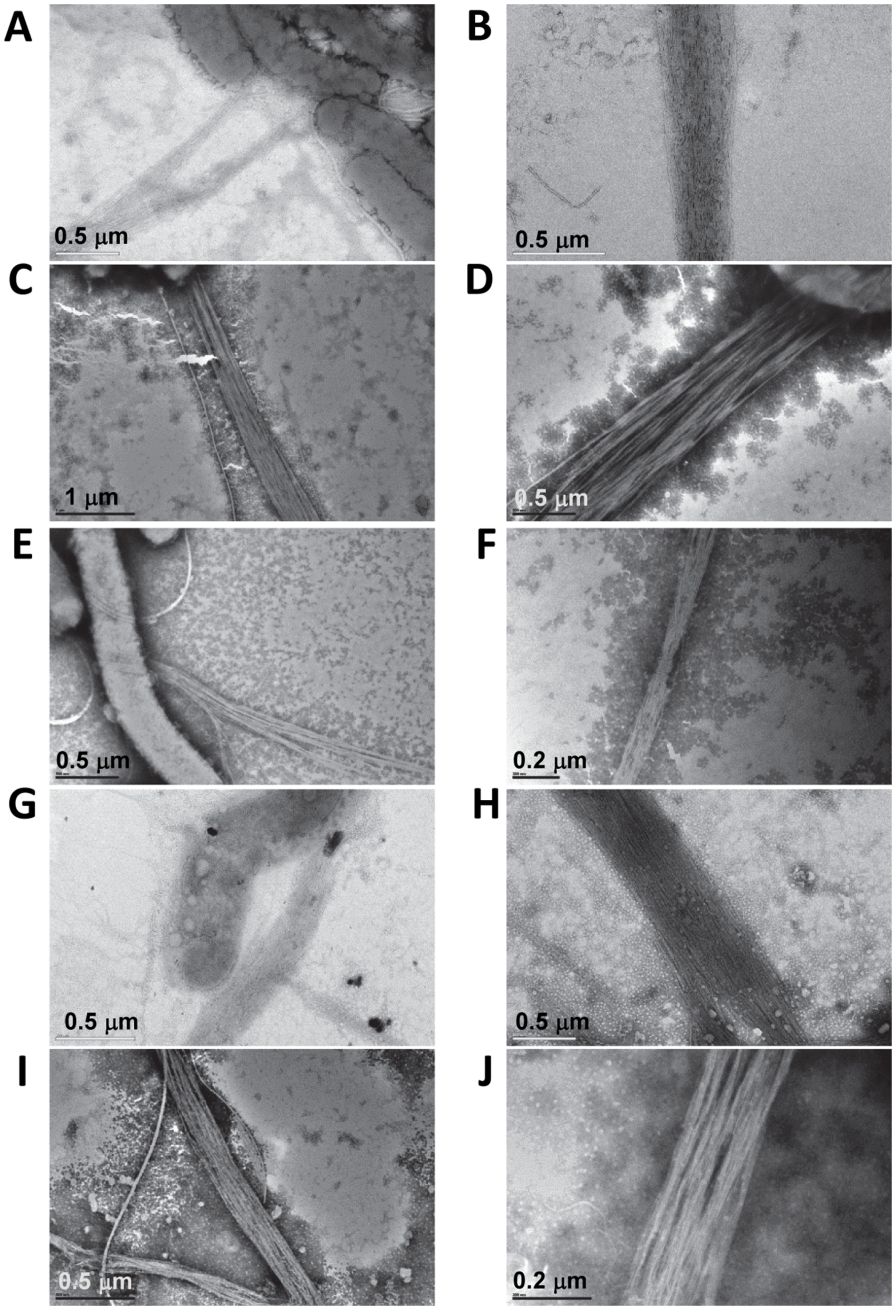
Effect of polyamines on TCP bundling. Representative TEM images showing rope-like bundles of TCP from cultures grown in the absence of polyamines (A,B) and in the presence of putrescine (C,D), cadaverine (E,F), spermidine (G,H) and spermine (I,J).

TCP from the classical strains of *V. cholerae* that have undergone autoagglutination have been shown to associate laterally into rope-like bundles [8], [12]. These pilus bundles are thought to be held together by electrostatic interactions between neighboring TcpA. Mutations in charged residues on TcpA have been shown to alter pilus bundling morphology by inhibiting the formation of rope-like bundles [12], [15]. The association of pilus bundles from neighboring cells is believed to mediate microcolony formation. Certain mutations in TcpA has been shown to produce pili that do not form the characteristic rope-like bundles as seen with wild-type TCP, but rather are wavy structures that do not bundle tightly [12], [15]. Since spermidine, spermine, and cadaverine did not reduce TCP biogenesis we hypothesized these molecules may be disrupting a step associated with pilus bundling. Using transmission electron microscopy (TEM), we examined whole cell cultures grown in the presence and absence of polyamines to determine whether these molecules inhibited pilus bundling. To maximize the effect on autoagglutination, polyamines were used at the concentration which inhibited agglutination by ≥70%. TEM analysis revealed that laterally associated rope-like bundles were found in all samples, indicating that polyamines have no effect on the pilus bundling morphology (Fig. 5).

Next, to determine if the functionality of the pili were compromised in the presence of polyamines, we performed phage transduction assays. TCP has been shown to be the high affinity receptor for the filamentous, lysogenic bacteriophage CTXφ, which carries the genes encoding the A and B subunits of cholera toxin [47]. To assess the functionality of TCP, we measured the ability of CTXφ to transduce *V. cholerae.* While the presence of polyamines did allow phage transduction to occur, the transduction frequency was reduced by 70–80% (Table 1), suggesting that functionality of the pili may be compromised under these conditions. Because no discernible morphological differences were observed in the pili in presence of polyamines, our data suggest that polyamines affect the functionality of the TCP by a mechanism that does not affect bundling.

## Discussion

### Effect of Polyamines on Autoagglutination and Levels of TcpA, TCP, and CT

In this work we have demonstrated that the polyamines putrescine, cadaverine, spermidine, and spermine have a dose-dependent inhibitory effect on the autoagglutination *of V. cholerae* O1 classical biotype. The effect was stronger with putrescine, spermidine, and spermine, all of which are polyamines that are synthesized by humans. Cadaverine, a bacterial polyamine, was the least effective in inhibiting agglutination. Combination of the all of the polyamines showed an effect similar to that of cadaverine only, suggesting that the presence of cadaverine may diminish effectiveness of the other three polyamines in inhibiting autoagglutionation.

Despite having a similar effect on autoagglutination, these polyamines display distinct and varying effects on other determinants of *V. cholerae* virulence. Putrescine and cadaverine caused a small reduction on the levels of TcpA, the major subunit of TCP which is responsible for the agglutination phenotype. Putrescine also led to a small but significant decrease in TCP biogenesis, which could be explained by the reduction of TcpA levels. The decrease in the availability of the major pilus subunit would inevitably lead to a reduction in the number of TCP present on the cell surface. However, cadaverine did not lead to a reduction in TCP levels. It is possible that despite the reduction of TcpA levels by cadaverine, the intracellular pool of TcpA was still adequate to support normal levels of TCP biogenesis. Spermidine caused an increase in TcpA as well as TCP levels under the conditions where it decreased autoagglutination. It is possible that spermidine that is imported by the cell enhances TcpA production whereas exogenous spermidine decreases autoagglutination. Regardless of the mechanisms of these opposing effects, the inhibitory effect of spermidine appeared to overcome its stimulatory effect. Spermine did not affect TcpA or TCP levels. These effects did not to correlate well with increases in osmolarity resulting from polyamine supplementation of the media. Our results suggest that the effect of putrescine on agglutination could partially be explained by a reduction in TcpA levels leading to lower amounts of pili on the cell surface; however, cadaverine, spermidine, and spermine reduce agglutination by a different mechanism.

We also tested the effect of polyamines on CT levels as genes encoding components of cholera toxin and TCP are coregulated by the transcriptional regulator ToxT. We hypothesized that because putrescine, cadaverine, and spermidine affected levels of TcpA in the cell, they could also affect CT levels. Surprisingly, only spermine, which did not affect TcpA levels, had a small effect on CT production. Certain environmental inputs have been shown to affect expression of the *tcp* operon and the *ctxAB* genes differently. For example, sodium cholate, a component of bile, modulates cholera toxin but not TcpA levels in a ToxT-independent manner [21]. Our results indicate that polyamines also have differential effects on the production of these two virulence factors. In addition, because osmolarity has been shown to affect CT and TcpA production similarly these results are also consistent with the hypothesis that increases in alone osmolarity cannot account for the effects of polyamines on virulence factor production.

### Pilus Structure and Function

Pilus bundling is necessary for autoagglutination of *V. cholerae*. TEM analysis showed that the polyamines did not interfere with pilus bundling, suggesting that these molecules disrupt autoagglutination by interfering with a step subsequent to pilus bundling. Consistent with our findings, it has been reported that the presence of rope-like bundles is not sufficient for autoagglutination as mutations in several charged residues of TcpA still allowed formation of laterally associated rope-like bundles yet significantly impaired autoagglutination [12], [15]. Also, it has recently been suggested that there is a hierarchy to pilus bundling which starts with pilus filaments from individual cells laterally associating to form higher ordered rope-like bundles [17]. These smaller bundles then associate with other bundles from neighboring cells to form larger supertwisted bundles. Since we were unable to discern these higher ordered supertwisted structures from single cell pilus bundles by regular TEM analysis, it is possible that polyamines may be disrupting these higher ordered, supertwisted structures. Thus, we hypothesize polyamines may be inhibiting autoagglutination by interacting directly with pili and disrupting the pili-pili interactions between neighboring cells which are thought to mediate autoagglutination. This interaction may be partially electrostatic due to the protonated amines on the polyamines. Our results also showed that the magnitude of the effect of polyamines depended on the type of polyamine used. Cadaverine was not as potent as the other polyamines in inhibiting autoagglutination. Cadaverine is the only polyamine used in this study that has a 5-carbon backbone whereas the other three polyamines either solely contain a 4-carbon backbone (putrescine) or have a diaminobutane moiety in part of the molecule (spermidine and spermine). It is possible that four carbons constitute optimum spacing between the two positive charges for optimum binding to the pili. The effect of polyamines on CTXφ transduction could also be explained by a similar inhibition of interaction of the phage with its binding site on TCP; however, this proposed interaction does not appear to be sensitive to the nature of the polyamine.

### Implications for Pathogenesis

Our results suggest that polyamines, in particular putrescine, spermidine, and spermine, in the human intestinal environment could modulate the infection caused by *V. cholerae* O1 classical strain by inhibiting autoagglutination. Polyamines have been reported to be in the mid-to-high micromolar range in the human intestinal lumen, which is below the concentration shown in this study to inhibit autoagglutination [48], [49]. This observation is consistent with the fact that *V. cholerae* can easily colonize most individuals if ingested in sufficient amounts. However, in order to colonize the small intestine, *V. cholerae* has to swim through the mucus layer to get to the intestinal villi and lumen concentrations of polyamines may differ significantly from levels of these molecules in the mucus layer. Intestinal microenvironments encountered by *V. cholerae* during infection have a significant effect on virulence gene expression. A recent study has shown that transcription of the *tcpA* gene is increased 30-fold in *V. cholerae* isolated from an epithelial cell surface/mucus layer fraction of rabbit ileal loops compared to mid-log phase cultures whereas it is increased only 3.5 fold in the lumen fraction [5]. The same study also showed that *tcpA* expression was highest in bacteria located within 0–5 µm of the epithelial surface and decreased in bacteria that were farther away. These results make polyamine levels in the immediate vicinity of the epithelial cell surface highly relevant.

Polyamines are found in millimolar amounts in cells and high amounts of polyamines have been detected in epithelial cells lining the intestinal crypts and the villi [50]. Therefore, large amounts of polyamines would be expected to be released into the intestinal crypts and the mucus layer as a result of the high turnover rate of intestinal epithelial cells [51]. These polyamines may be concentrated in the mucus layer and act as a barrier that decreases microcolony formation and discourages colonization of the intestines by *V. cholerae.* Studies in animal models are needed to test this hypothesis and the effects of polyamines on *V. cholerae* virulence *in vivo.*

## Supporting Information

Figure S1 **Effect of mixed polyamines on** ***V. cholerae*** **autoagglutination.** *V. cholerae* was cultured in the presence of increasing concentrations of a mix of equivalent amounts of putrescine, cadaverine, spermidine and spermine under virulence factor inducing conditions. Each polyamine was added to contribute 0.15, 0.3, and 0.45 mmoles of positive charges to the media. Negative controls were grown in media with a pH of 8.5 at 37°C. Sedimentation index was calculated as 1– (Final A_595_/Initial A_595_). Each sample is the average of three biological replicates with error bars representing the standard deviation. Pair-wise Student’s t-tests were performed to compare SI’s of cultures grown in the absence of polyamines to cultures grown with each different polyamine Stars indicate p<0.01.
(TIF)
