# Supplementary figures and images for: Effects of Polyamines on Vibrio cholerae Virulence Properties
Source: PLoS One. 2013 Apr 10;8(4):e60765. doi: 10.1371/journal.pone.0060765 (PMC3622680; doi:10.1371/journal.pone.0060765)

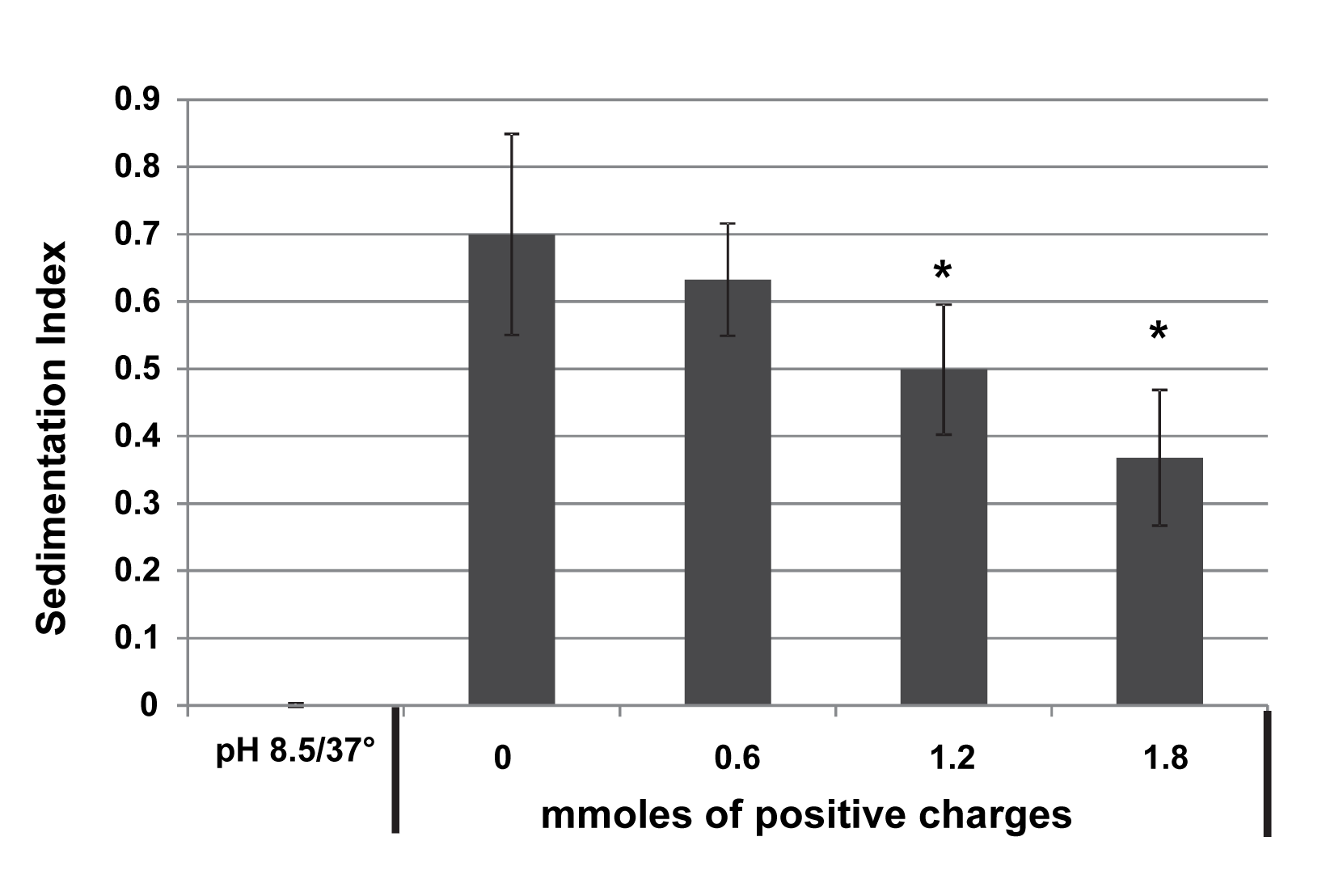

Supplement: Figure S1 — Effect of mixed polyamines on V. cholerae autoagglutination. V. cholerae was cultured in the presence of increasing concentrations of a mix of equivalent amounts of putrescine, cadaverine, spermidine and spermine under virulence factor inducing conditions. Each polyamine was added to contribute 0.15, 0.3, and 0.45 mmoles of positive charges to the media. Negative controls were grown in media with a pH of 8.5 at 37°C. Sedimentation index was calculated as 1– (Final A595/Initial A595). Each sample is the average of three biological replicates with error bars representing the standard deviation. Pair-wise Student’s t-tests were performed to compare SI’s of cultures grown in the absence of polyamines to cultures grown with each different polyamine Stars indicate p<0.01. (TIF) [file pone.0060765.s001.tif]
